# A large-scale norovirus outbreak associated with kimchi consumption across multiple schools in a Korean city in 2024

**DOI:** 10.4178/epih.e2025057

**Published:** 2025-10-03

**Authors:** Eunkyung Park, Sumi Cho, Eunyoung Kim, Jung Im Park, Ju-Hyung Lee, Jin Gwack

**Affiliations:** 1 Division of Infectious Disease Response, Honam Regional Center for Disease Control and Prevention, Gwangju, Korea; 2 Jeonbuk State Center for Infectious Disease Control and Prevention, Jeonju, Korea; 3 Department of Preventive Medicine, Jeonbuk National University Medical School, Jeonju, Korea; 4 Institute for Medical Sciences, Jeonbuk National University, Jeonju, Korea

**Keywords:** Norovirus, Disease outbreak, Foodborne diseases

## Abstract

**OBJECTIVES:**

Norovirus is a major global concern for foodborne outbreaks. We investigated a large-scale norovirus outbreak in a Korean city to identify the source of infection and implement control measures.

**METHODS:**

A retrospective cohort study was conducted. Data were collected from questionnaires and laboratory test results, along with an environmental investigation. Human and environmental samples were obtained.

**RESULTS:**

The overall attack rate was 20.2%, with 862 cases among 4,276 individuals exposed across 24 affected schools. Norovirus GII.17 was detected in symptomatic individuals, asymptomatic food handlers, and cabbage kimchi products. Kimchi consumption was significantly associated with illness (relative risk, 3.85; 95% confidence interval [CI], 3.12 to 4.74) and was confirmed as the outbreak source. In multivariate logistic regression, both high school status (odds ratio [OR], 1.49; 95% CI, 1.11 to 2.00) and kimchi consumption (OR, 5.56; 95% CI, 4.24 to 7.30) emerged as significant risk factors.

**CONCLUSIONS:**

Kimchi contamination likely occurred either through ingredients or food handlers during the manufacturing process. This study provides key insights for prevention and rapid response to norovirus outbreaks, emphasizing the vulnerability of school food services. We highlight the importance of stringent food safety practices and routine training for food handlers, particularly in manufacturing facilities, given the role of asymptomatic carriers.

## GRAPHICAL ABSTRACT

[Fig f3-epih-47-e2025057]

## Key Message

An epidemiological investigation into a large-scale norovirus outbreak associated with 24 schools identified contaminated kimchi as the likely source. The outbreak underscores the critical need for proper hygiene management of food ingredients and effective control of asymptomatic food handlers during the preparation of mass meals in institutional settings such as school food services.

## INTRODUCTION

Norovirus is a leading cause of acute gastroenteritis and a major contributor to waterborne and foodborne outbreaks worldwide, affecting both developed and developing countries [1,2]. The globally predominant genotype is GII.4, including in Korea [2,3]. Although outbreaks generally occur with winter seasonality [4], norovirus remains the most common cause of food poisoning year-round in Korea [3]. Transmission frequently occurs through contaminated food and water, including ready-to-eat products, raw shellfish (such as oysters), and fresh produce [5-7]. The virus is also highly contagious through person-to-person contact [8], as even a small number of viral particles can cause illness [8,9]. In healthy adults, norovirus infections are usually acute and self-limited, with symptoms such as diarrhea, vomiting, and abdominal pain resolving within 3 days in patients older than 12 years [10]. However, immunocompromised individuals are more vulnerable to infection, and globalization of the food industry has increased the potential for large-scale outbreaks [9]. Because of its low infectious dose [8], prompt recognition of outbreak characteristics is critical for effective response and prevention.

Norovirus outbreaks demand rapid responses and strict control measures to reduce their impact on susceptible populations. They present a considerable public health challenge, particularly in semi-enclosed environments such as schools, healthcare facilities, and cruise ships [11]. Large-scale school outbreaks can have severe repercussions, affecting students and staff, disrupting educational activities, and generating community-wide health concerns [5-7,12].

On Tuesday, July 2, 2024, a suspected foodborne outbreak was reported across multiple schools in a small Korean city. This triggered an epidemiological investigation to identify the source of infection, determine the outbreak’s scope, and implement control measures. The investigation focused on both the affected schools and the food manufacturing facility that supplied the suspected contaminated product. This study reports the findings of a retrospective cohort investigation that characterized the epidemiological features of this large-scale norovirus outbreak. Furthermore, it details the investigation process, presents key findings regarding the outbreak’s source and extent, and discusses implications for preventing future norovirus outbreaks in school settings.

## MATERIALS AND METHODS

### Outbreak identification

In the small city of Namwon, Jeonbuk State, Korea, many students from different schools presented to the emergency room within a few hours on the night of Tuesday, July 2, 2024, with gastrointestinal symptoms including diarrhea, vomiting, fever, and abdominal pain. The unusually high number of student visits prompted medical staff to report a suspected waterborne and foodborne disease outbreak to the local public health authority on the same day. Local health authorities immediately initiated an epidemiological investigation and sample collection. Specimens from symptomatic patients were obtained in the emergency room. The outbreak response was carried out by the local public health center, the Jeonbuk State provincial office, the Jeonbuk State Center for Infectious Disease Control and Prevention, and the Honam Regional Center for Disease Control and Prevention. The Korea Disease Control and Prevention Agency (KDCA) conducts national surveillance for notifiable infectious diseases, including norovirus, which is classified as a category IV disease under sentinel surveillance. In addition, local public health authorities require the reporting of any event in which 2 or more individuals develop gastrointestinal symptoms after consuming the same food or water [13]. This reporting system allows early outbreak detection, facilitates monitoring of outbreak trends, and supports timely implementation of control measures [14].

### Study design and case definition

All students and staff from the affected schools were included in this study. A retrospective cohort study design was adopted to identify the source of infection and guide control measures. A case was defined as an individual presenting with more than 1 episode of diarrhea or vomiting, or with laboratory-confirmed norovirus infection, after consuming a school meal on July 1, 2024 or July 2, 2024, among students, staff, and food handlers at the affected schools. The study used web-based questionnaires standardized by the KDCA, which were modified through the Jeonbuk State Center for Infectious Disease Control and Prevention. Students and staff were required to complete online self-administered questionnaires to collect demographic information, symptom details, and food intake history during the estimated exposure period (June 27 to July 2, 2024), based on school meal records.

### Epidemiological investigation

The initial patients presenting to the emergency room were students from multiple schools in the city. To identify potential exposure sources, we reviewed school information, including names, scheduled events, the layout and location of water purifiers, and water supply systems. Additional data on symptomatic individuals were obtained from the National Education Information System (NEIS) and from schools, along with school food service menus, and were incorporated into the questionnaires. Kimchi was identified as the only uncooked food item served across all affected schools. Therefore, the investigation centered on the kimchi manufacturing facility and the school food service supporting center. Staff at the kimchi manufacturing facility, including food handlers, were interviewed in person and by telephone to trace possible transmission routes and assess epidemiological risk factors.

Environmental investigations were carried out both at the kimchi facility and at schools reporting more than 10 symptomatic individuals. Several school food service workers, including nutrition teachers and dietitians, were interviewed during on-site inspections. Inspections covered kitchens, food service areas, and surrounding environments to identify potential contamination sources of the causative pathogen.

### Sample collections and genetic analysis

From July 2 to July 12, a total of 124 human biological specimens and 223 environmental specimens were collected by local public health officials. Human samples included 60 rectal swabs, 2 stool samples, and 1 vomitus sample from symptomatic individuals. Samples from asymptomatic individuals were collected from 45 school food handlers and 16 staff members at the kimchi manufacturing facility. Environmental samples included 93 preserved school food items, 36 environmental surfaces from kitchens, 35 kitchen utensils, 6 kimchi products from the facility, 5 raw food ingredients, and 48 water samples, including groundwater from the school food service supporting center.

Human specimens were tested for 16 bacterial and 5 viral pathogens, and environmental specimens were tested for 18 bacterial and 7 viral pathogens. Laboratory analyses were primarily performed at the Jeonbuk State Institute of Health and Environment Research, following the Methods for the Detection of Foodborne Pathogens issued by the Ministry of Food and Drug Safety. Norovirus-positive samples with adequate Ct values underwent genotyping by KDCA for human samples and by the Ministry of Food and Drug Safety for food samples.

### Statistical analysis

We collected data from questionnaires, including demographic and clinical information, symptom profiles, food intake history, and laboratory results. Attack rates (ARs) among exposed and unexposed groups were compared by calculating risk ratios (RRs) with 95% confidence intervals (CIs). To examine associations between risk factors and illness, multivariate logistic regression was performed to estimate odds ratios (ORs) with 95% CIs, including variables such as school type and kimchi consumption in school food services. Descriptive statistics were analyzed using the chi-square test or Fisher’s exact test for categorical variables and the Kruskal–Wallis test for continuous variables, with statistical significance defined as p-value <0.05. Data entry and cleaning were carried out using Microsoft Excel 2019 (Microsoft, Redmond, WA, USA) for Windows, and statistical analyses were performed using R version 4.3.1 (R Foundation for Statistical Computing, Vienna, Austria).

### Ethics statement

This study was approved by the Institutional Review Board (IRB) of Jeonbuk National University Hospital (IRB No. 2025-07-053). Because this outbreak investigation was conducted as part of an urgent public health response under the Infectious Disease Control and Prevention Act (Article 4) and the Enforcement Rule of the Bioethics and Safety Act (Article 33), the IRB waived the requirement for informed consent.

## RESULTS

### Characteristics of the outbreak

Among the 50 schools in the city, 42 received contaminated kimchi, and 24 (57.1%) reported more than 2 individuals with gastrointestinal symptoms. Of the 4,276 individuals exposed in these 24 schools, 1,920 completed questionnaires, yielding a 44.9% response rate. Of the 1,032 individuals who self-reported gastrointestinal symptoms, 862 met the case definition, including 42 laboratory-confirmed cases. The overall AR was 20.2% (862/4,276). By school type, elementary schools had the highest AR at 25.5% (342/1,341 cases), followed by high schools at 21.7% (376/1,735 cases) (Table 1).

The sex distribution of cases was comparable, with males accounting for 50.4% and females 49.6% (p=0.038). Students represented the majority of cases at 84.7% (730 cases), while staff accounted for 15.3% (132 cases), including 1.9% (16 cases) among food handlers. All residents of boarding schools, classified as high school students, received meals 3 times daily, and 91.5% of high school cases (344 of 376) occurred in these boarding schools. The most frequent symptoms were diarrhea (75.0%), vomiting (71.9%), and abdominal pain (66.9%). Among respondents providing information on diarrhea, 27.4% (144/525) reported more than 5 episodes. Similarly, among those reporting vomiting, 27.7% (172/492) experienced more than 5 episodes (Table 2).

**Figure 1.**
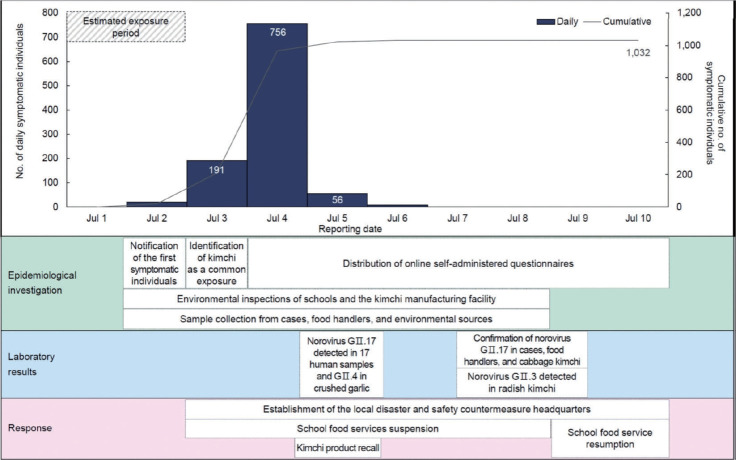
Timeline of events and public health responses during a norovirus outbreak in Korea, July 2024. The timeline illustrates the number of self-reported symptomatic individuals by reporting date, along with key public health responses. Blue bars represent the daily number of symptomatic individuals (left axis), while the gray line indicates the cumulative number of symptomatic individuals (right axis). Boxes below the timeline summarize public health responses, categorized into three sections: epidemiological investigations, laboratory results, and responses.

**Figure 2.**
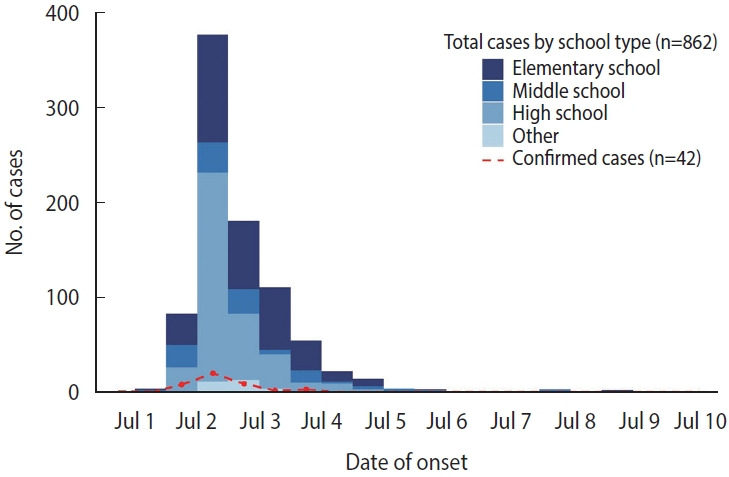
Epidemic curve of a norovirus outbreak in Korea, July 2024. The epidemic curve presents the number of total cases stratified by school type, along with laboratory-confirmed cases. Bars represent the total number of cases, while the red dotted line indicates the number of laboratory-confirmed cases. The x-axis is divided into half-day (12-hour, AM/PM) intervals.

The median incubation period was 34.0 hours (interquartile range [IQR], 30.0-47.0), with elementary schools showing a median of 37.0 hours (IQR, 30.4-55.0) (Table 2, Supplementary Material 1). Figure 1 shows the timeline of self-reported symptomatic vehicle and limit further spread. Based on comprehensive data, the first case occurred at 16:00 on July 1, and the outbreak concluded on July 14. The epidemic curve peaked between noon and midnight on July 2, consistent with a point-source outbreak caused by a single common exposure (Figure 2). Considering the epidemic curve, the known norovirus incubation period (10-50 hours), and the suspension of school meals after July 3, the most probable exposure period was lunch on July 1. However, for the 4 schools that did not provide lunch on July 1, the estimated exposure period was July 2.

### Laboratory investigation

Norovirus GII.17 was confirmed as the causative agent of the outbreak, with contaminated cabbage kimchi identified as the likely vehicle of transmission. A total of 124 human specimens were collected, including samples from 63 symptomatic cases, 45 school food handlers, and 16 food handlers at the kimchi manufacturing facility. Of these, 48 specimens tested positive for norovirus using reverse transcription-polymerase chain reaction. Positive results were obtained from 33 symptomatic students and staff, 9 asymptomatic school food handlers (cases), and 6 asymptomatic food handlers at the kimchi facility (non-cases). The overall human positivity rate was 38.7%, comprising 52.4% (33/63) among symptomatic cases, 20.0% (9/45) among school food handlers, and 37.5% (6/16) among facility handlers. Of the 48 positive human samples, 40 were genotyped as GII.17. Among 223 environmental specimens, including food, water, kitchen utensils, and environmental surfaces, norovirus GII strains were identified in kimchi products and food ingredients. Specifically, GII.17 was detected in 2 cabbage kimchi samples preserved for school meals at a high school. In contrast, GII.3 was confirmed in a radish kimchi sample stored in a refrigerator at another high school, and GII.4 was detected in crushed garlic from the kimchi facility. Importantly, the viral genotypes found in symptomatic and asymptomatic cases, food handlers at the facility, and 2 cabbage kimchi samples from school meals were all GII.17 (Table 3).

### Epidemiological investigation

Based on laboratory results from both human and kimchi samples, field investigations were conducted at the kimchi manufacturing facility and the local school food service supporting center. A waterborne outbreak was ruled out, as no water-related events had been reported in the city in the preceding months. The affected schools procured food ingredients and kimchi through a single local school food service center, which was managed by the local office of education to reduce costs and simplify contracting [15]. A total of 42 schools received kimchi supplied by 1 manufacturing facility through this center. At the food service center, kimchi packages were delivered directly to schools without being opened. Groundwater testing revealed no evidence of norovirus contamination. Consequently, the investigation focused on identifying the mechanism of norovirus contamination in kimchi products during manufacturing. Neither shellfish such as oysters nor groundwater, both commonly linked to norovirus outbreaks, were used in kimchi preparation. Instead, cabbage was washed and salted with tap water before being mixed with seasonings and ingredients such as garlic and ginger. According to the facility manager, the mixing process followed Hazard Analysis and Critical Control Points (HACCP) guidelines, and ingredients were treated with diluted chlorine. Observed hygiene standards appeared to be within acceptable levels.

To determine the likely contamination route, personal questionnaires and environmental inspection findings were reviewed. Interviews confirmed that food handlers who tested positive for norovirus were asymptomatic yet directly involved in production tasks, including washing and salting cabbage, preparing spices, and mixing kimchi seasoning. The median age of food handlers was 66.7 years, and most were female (83.3%, 5/6). As staff members commonly consumed kimchi during lunch, they were presumed to have been exposed through ingestion. One food handler reported using groundwater at home, but no household members had gastrointestinal symptoms. The exact manufacturing date of the contaminated kimchi could not be confirmed but was estimated to be 2 days to 3 days before distribution. No evidence of contamination by other pathogens was identified during the suspected period of production. Overall, these epidemiological findings indicate that norovirus contamination occurred during cabbage kimchi manufacturing, most likely through asymptomatic food handlers.

### Analytical epidemiology

A retrospective cohort study was conducted to identify the primary risk factor for the outbreak. Among the 1,920 individuals who completed questionnaires, 1,613 provided detailed information on common food intake. Of these, 839 individuals reported consuming cabbage kimchi in school cafeterias, while 774 reported no exposure to kimchi during the estimated exposure period. The AR among the exposed group was 44.2%, compared to 11.5% in the non-exposed group. The overall RR of illness associated with cabbage kimchi consumption was 3.85 (95% CI, 3.12 to 4.74). By school type, the highest RR was observed in middle schools (5.27, 95% CI, 3.40 to 8.16) (Table 4). Logistic regression analysis further showed that high school students and staff had a significantly higher OR of 1.49 (95% CI, 1.11 to 2.00) compared with elementary school members (reference group). Notably, individuals who consumed kimchi in school food services exhibited the strongest association with illness, with an OR of 5.56 (95% CI, 4.24 to 7.30), underscoring the critical role of kimchi consumption in this outbreak (Table 5).

### Responses to the outbreak

After receiving notification of the suspected outbreak, public health authorities collaborated with the local office of education to coordinate responses in affected schools and monitor daily reports of symptomatic individuals. The hospital managed the increased number of emergency room visits by designating additional treatment spaces. To reduce further transmission, symptomatic food handlers from both the affected schools and the kimchi manufacturing facility were temporarily excluded from work duties. Following laboratory confirmation of contamination, the facility voluntarily recalled the implicated kimchi products. From July 3 to July 8, school food services were suspended in the 24 affected schools to prevent additional exposures. During this period, alternative foods such as dairy and bakery products were provided. Before cafeterias reopened, all food ingredients, including kimchi, were discarded, and kitchen environments were disinfected. When school meals resumed, only heated dishes were served, in accordance with public health authority recommendations. In response to the scale of the outbreak, a local Disaster and Safety Countermeasure Headquarters was temporarily established to ensure rapid coordination. The headquarters issued a crisis alert, raising the regional level from “Attention” (level 1) to “Caution” (level 2) between July 3, 2024 and July 10, 2024. Updates on the outbreak’s progression were provided through press releases, and the headquarters coordinated response measures across institutions.

## DISCUSSION

The extensive norovirus outbreak affected 57.1% (24/42) of schools in Namwon, a small Korean city, resulting in 1,032 self-reported symptomatic individuals. This large-scale outbreak is reminiscent of the 2006 norovirus outbreak in Korea, which affected approximately 2,500 individuals across 26 schools [3]. Since the 2000s, norovirus has remained the leading cause of foodborne outbreaks in Korea, excluding those of unknown origin, regardless of the coronavirus disease 2019 (COVID-19) pandemic [3,16,17]. School food services have been implicated in nearly one-third of norovirus outbreaks [3]. The overall AR across school types was 20.2% (862/4,276), ranging from 25.5% in elementary schools to 10.6% in middle schools (Table 1). Students accounted for most cases (84.7%), while staff represented 15.3%, including 1.9% (16 cases) among food handlers (Table 2). Although the AR was lowest in middle schools, the RR of kimchi consumption was highest in this group (RR, 5.27; 95% CI, 3.40 to 8.16) (Table 4). Middle schools also demonstrated the highest questionnaire response rate (56.7%) and case reporting rate (77.2%), which may have influenced these findings. In contrast, high school members were more likely to develop norovirus illness. Both kimchi consumption (OR, 5.56; 95% CI, 4.24 to 7.30) and attendance at high school (OR, 1.49; 95% CI, 1.11 to 2.00) were significantly associated with illness (Table 5). The provision of 3 daily meals in boarding schools, where 91.5% (344/376) of high school cases occurred, likely increased exposure and contributed to the higher risk of illness in this group.

The majority of symptomatic cases presented with gastrointestinal symptoms, including diarrhea (75.0%), vomiting (71.9%), and abdominal pain (66.9%). Diarrhea was particularly frequent among middle and high school students (80.7 and 80.6%, respectively). Abdominal pain was also common in high schools (73.7%) and middle schools (71.1%). High school students reported a notable prevalence of nausea (70.5%). These findings suggest that students in boarding schools, consuming 3 meals daily, may have experienced higher viral loads, contributing to severe symptoms, consistent with prior research [18]. By contrast, vomiting and fever were more frequently reported in elementary schools. The median frequency of both diarrhea and vomiting was 3.0 episodes, with diarrhea most frequent in high schools and vomiting most frequent in elementary schools (Table 2). These patterns align with earlier studies indicating that vomiting and fever are more commonly experienced by children under 15 years [19].

The combined epidemiological, microbiological, and environmental evidence strongly indicates that kimchi consumption was the primary vehicle of transmission in this outbreak. The epidemic curve was consistent with a point-source outbreak, peaking 24 hours to 36 hours after exposure (Figure 2). Epidemiological findings revealed that all 24 affected schools received food supplies from a single school food service center. Kimchi consumption was associated with a 3.85-fold higher risk of illness. Multivariate logistic regression identified both high school attendance (OR, 1.49; 95% CI, 1.11 to 2.00) and kimchi consumption (OR, 5.56; 95% CI, 4.24 to 7.30) as risk factors (Table 5). Microbiological testing confirmed that norovirus GII.17 detected in cabbage kimchi samples from a high school matched the strain identified in symptomatic cases and asymptomatic food handlers at the kimchi facility (Table 3). Kimchi was the only common food consumed by all affected groups, and laboratory evidence supported its role in transmission.

Although the precise route of contamination could not be determined, both contaminated food ingredients and asymptomatic food handlers are plausible sources. Notably, 37.5% (6/16) of the staff tested positive for norovirus but were asymptomatic, having consumed the same kimchi. This finding is consistent with previous studies showing that asymptomatic food handlers can shed high viral loads and play a critical role in norovirus transmission [20-23]. Our findings underscore the importance of testing asymptomatic food handlers during outbreaks and recognizing their key role in viral spread [20-22]. To prevent transmission from asymptomatic carriers, alongside monitoring symptomatic individuals, it is essential to strengthen management and training of food handlers. Standard food safety practices—particularly strict adherence to hand hygiene before and after meals and after restroom use—should be reinforced to reduce the risk of norovirus transmission.

Kimchi contamination may also have occurred through contaminated ingredients. However, environmental inspections and laboratory testing at the kimchi manufacturing facility revealed no evidence of contamination pathways, either from ingredients or food handlers. Kimchi, a fermented side dish widely consumed in Korean meals, is produced in bulk and distributed to many people through institutional food services, including schools and workplaces. The large-scale exposure associated with group meal services likely amplified the impact of consuming contaminated kimchi. Previous norovirus outbreaks in Korea have identified kimchi contaminated by groundwater or food handlers as sources of infection [6,23,24]. Norovirus is stable at pH 3-7 but can degrade under highly acidic conditions [25,26]. Prior studies suggest that its physicochemical properties—such as organic acid production and reduced pH during fermentation—may decrease viral titers of both human and murine norovirus depending on temperature and duration. However, fermentation does not guarantee complete viral inactivation [27,28]. To prevent contamination during production, even throughout fermentation [27,28], it is critical to reinforce food safety practices, particularly in the handling of raw ingredients at kimchi manufacturing facilities supplying group meals, in order to reduce the risk of future outbreaks.

This outbreak highlights the substantial public health risks posed by foodborne pathogens in institutional settings, especially schools. The extensive scale and rapid spread among students, staff, and food handlers underscore the importance of strict food safety protocols in kimchi production and distribution, particularly in school food services. The involvement of asymptomatic food handlers in kimchi preparation demonstrates the risk of silent transmission within food establishments [20-22]. In addition, the outbreak illustrates the challenges faced by public health authorities in managing large-scale incidents, emphasizing the need for close coordination between government ministries responsible for health, education, and food service management.

This study has several limitations. First, the retrospective cohort design relied on collected data that may be subject to recall bias, particularly regarding food consumption history. The questionnaire response rate was 44.9%, and non-participants may have differed in exposure or risk factors. Second, due to limited availability of human specimens, the proportion of laboratory-tested cases varied from 0% to 10.5% across schools. Increasing awareness of early specimen collection and enhancing cooperation through clear communication could improve data reliability. Third, although norovirus was confirmed in both human and food specimens, further investigation of the food supply chain—particularly garlic—was not undertaken due to practical constraints. The precise mechanism of contamination during kimchi production therefore remains uncertain. Although water contamination near the kimchi facility was considered, a detailed investigation of pipelines was not performed. Moreover, although no community outbreaks were reported through the national outbreak surveillance system, unreported symptomatic individuals or undetected clusters may have been missed. Strengthening collaborative, joint investigations by relevant ministries and authorities will improve the effectiveness of outbreak responses.

This large-scale outbreak demonstrates the considerable vulnerability of school food services. Evidence strongly implicates contaminated cabbage kimchi as the vehicle of transmission, likely facilitated by asymptomatic food handlers at the facility. Public health measures—including enhanced food safety protocols and strengthened surveillance—are critical to preventing similar outbreaks. Food handlers should receive routine training and strictly adhere to food safety guidelines during kimchi production, and symptomatic individuals must be prohibited from handling food until fully recovered [29,30]. Prompt responses and coordinated efforts between health authorities, food producers, and schools are essential to mitigating the impact of outbreaks. Finally, further research should examine environmental conditions within kimchi facilities and external factors contributing to food contamination, as well as the role of asymptomatic food handlers in both waterborne and foodborne outbreaks. This study underscores the necessity of rigorous food safety measures and proactive surveillance to prevent and manage large-scale norovirus outbreaks in school settings.

## Figures and Tables

**Figure f3-epih-47-e2025057:**
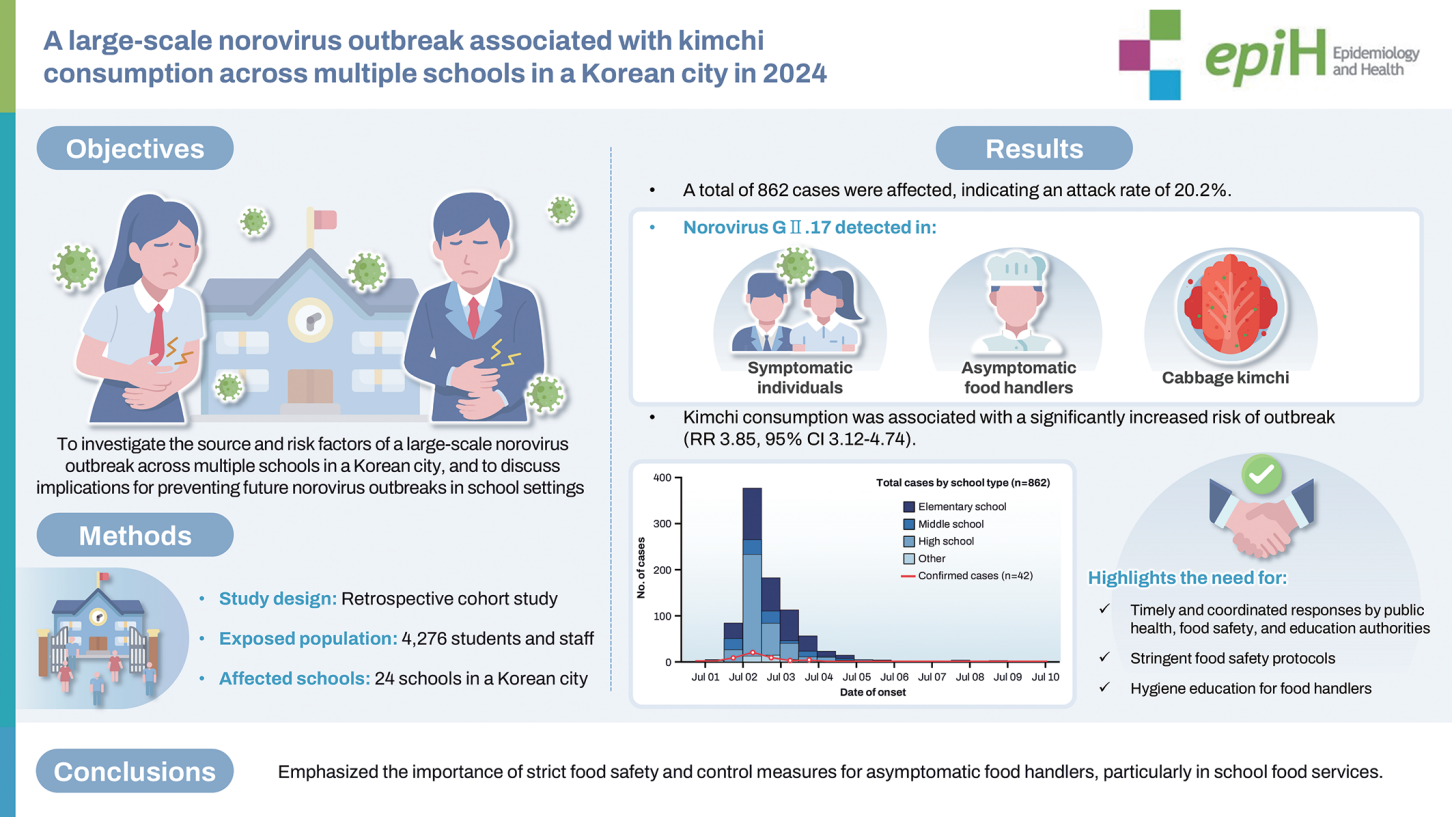


**Table 1.** Attack rates by school type during a norovirus outbreak in a city in Korea

Type of school	Schools	Exposed individuals	Cases	Attack rate (%)
Total	24	4,276	862	20.2
Elementary school	10	1,341	342	25.5
Middle school	7	1,076	114	10.6
High school	6	1,735	376	21.7
Other	1	124	30	24.2

Values are presented as number.

**Table 2.** Characteristics of cases by school type during a norovirus outbreak in a city in Korea

Characteristics	Total (n=862)	Elementary school (n=342)	Middle school (n=114)	High school (n=376)	Other (n=30)	p-value
Sex (n=679)^1^	679	241	94	323	21	0.038
Male	342 (50.4)	107 (44.4)	47 (50.0)	180 (55.7)	8 (38.1)	
Female	337 (49.6)	134 (55.6)	47 (50.0)	143 (44.3)	13 (61.9)	
Occupation						<0.001^2^
Student	730 (84.7)	283 (82.7)	83 (72.8)	347 (92.3)	17 (56.7)	
Staff	132 (15.3)	59 (17.3)	31 (27.2)	29 (7.7)	13 (43.3)	
Boarding school						<0.001
Yes	344 (39.9)	0 (0)	0 (0)	344 (91.5)	0 (0)	
No	518 (60.1)	342 (100)	114 (100)	32 (8.5)	30 (100)	
Symptom^3^				303 (80.6)		
Diarrhea	646 (75.0)	232 (68.0)	92 (80.7)		19 (63.3)	<0.001
Vomiting	619 (71.9)	275 (80.6)	73 (64.0)	246 (65.4)	25 (83.3)	<0.001
Abdominal pain	576 (66.9)	203 (59.5)	81 (71.1)	277 (73.7)	15 (50.0)	0.002
Nausea	533 (61.9)	191 (56.0)	60 (52.6)	265 (70.5)	17 (56.7)	0.001
Fever	408 (47.4)	183 (53.7)	47 (41.2)	167 (44.4)	11 (36.7)	0.012
Chill	267 (31.0)	104 (30.5)	25 (21.9)	127 (33.8)	11 (36.7)	0.106
Episode of symptom						
Diarrhea	3.0 (2.0-5.0)	3.0 (2.0-4.0)	3.0 (2.0-5.0)	3.0 (2.0-5.0)	3.5 (2.3-5.0)	<0.001
Vomiting	3.0 (2.0-5.0)	4.0 (2.0-6.0)	3.0 (2.0-4.0)	3.0 (2.0-5.0)	3.0 (2.0-4.0)	<0.001
Incubation period (hr)	34.0 (30.0-47.0)	37.0 (30.4-55.0)	34.0 (14.0-55.8)	33.0 (30.0-40.0)	38.0 (33.0-43.9)	<0.001

Values are presented as number (%) or median (interquartile range).

1 Sex was reported by 679 of 862 respondents; Subtotals are shown only for the sex category.

2 The Fisher exact test was conducted.

3 Respondents could select multiple symptoms, so the total percentage may exceed 100%.

**Table 3.** Laboratory results of norovirus in human, food, water, and environmental samples

Subjects	Schools		Facilities^1^		Genotypes of norovirus (no. of sequencing samples)
	Total	Norovirus-positive	Total	Norovirus-positive	
Total (human) (n=124)	108	42 (38.9)	16	6 (37.5)	GII.17 (40)
Symptomatic	63	33 (52.4)	-	-	GII.17 (33)
Asymptomatic	45	9 (20.0)	16	6 (37.5)	GII.17 (7)
Total (food, water, and environment) (n=223)	194	3 (1.5)	29	1 (3.3)	GII.17 (2), GII.3 (1), GII.4 (1)
Preserved school meals	93	3 (3.2)	-	-	GII.17 (2 cabbage kimchi), GII.3 (1 radish kimchi)
Kimchi products before distribution	-	-	6	0 (0)	N/A
Food ingredients	-	-	5	1 (20.0)	GII.4 (1 crushed garlic)
Cooking water	2	0 (0)	3	0 (0)	N/A
Tap water		0 (0)	2	0 (0)	N/A
Drinking water	40	0 (0)	-	-	N/A
Kitchen utensils	31	0 (0)	4	0 (0)	N/A
Surfaces	27	0 (0)	9	0 (0)	N/A

Values are presented as number or number (%). N/A, not available.

1 Facilities refer to the kimchi manufacturing facility and the local school food service supporting center.

**Table 4.** RR of cabbage kimchi consumption during the estimated exposure period in the norovirus outbreak across 24 schools^1^

Type of school	Exposed			Non-exposed			RR (95% CI)	p-value
	Total (n)	Cases (n)	AR (%)	Total (n)	Cases (n)	AR (%)		
Total	839	371	44.2	774	89	11.5	3.85 (3.12, 4.74)	<0.001
Elementary school	380	177	46.6	170	26	15.3	3.05 (2.10, 4.41)	<0.001
Middle school	205	64	31.2	405	24	5.9	5.27 (3.40, 8.16)	<0.001
High school	195	110	56.4	189	38	20.1	2.81 (2.06, 3.82)	<0.001
Other	59	20	33.9	10	1	10.0	3.39 (0.51, 2.51)	0.263

RR, relative risk; AR, attack rate; CI, confidence interval.

1 These results were analyzed for 1,613 exposed individuals, including 460 cases, who responded to the questionnaires.

**Table 5.** Multivariable regression analysis of factors associated with norovirus infection during the outbreak^1^

Variables	OR (95% CI)	p-value
Type of school		
Elementary school	1.00 (reference)	
Middle school	0.46 (0.34, 0.63)	<0.001
High school	1.49 (1.11, 2.00)	0.008
Other	0.58 (0.33, 1.01)	0.054
Consumption of kimchi served in school food services		
No	1.00 (reference)	
Yes	5.56 (4.24, 7.30)	<0.001

OR, odds ratio; CI, confidence interval.

1 This result was analyzed for 1,613 respondents among the 4,276 exposed individuals; Multivariable logistic regression included type of school and history of kimchi consumption in school food services.

## Supplementary materials

Supplementary Material 1. Distribution of incubation periods of cases of norovirus during the outbreak, stratified by (A) school type and (B) occupation
