# Supplementary material for: A large-scale norovirus outbreak associated with kimchi consumption across multiple schools in a Korean city in 2024
Source: Epidemiol Health. 2025 Oct 3;47:e2025057. doi: 10.4178/epih.e2025057 (PMC12869137; doi:10.4178/epih.e2025057)

Supplementary Material 1. Distribution of incubation periods of cases of norovirus during the outbreak, stratified by (A) school type and (B) occupation


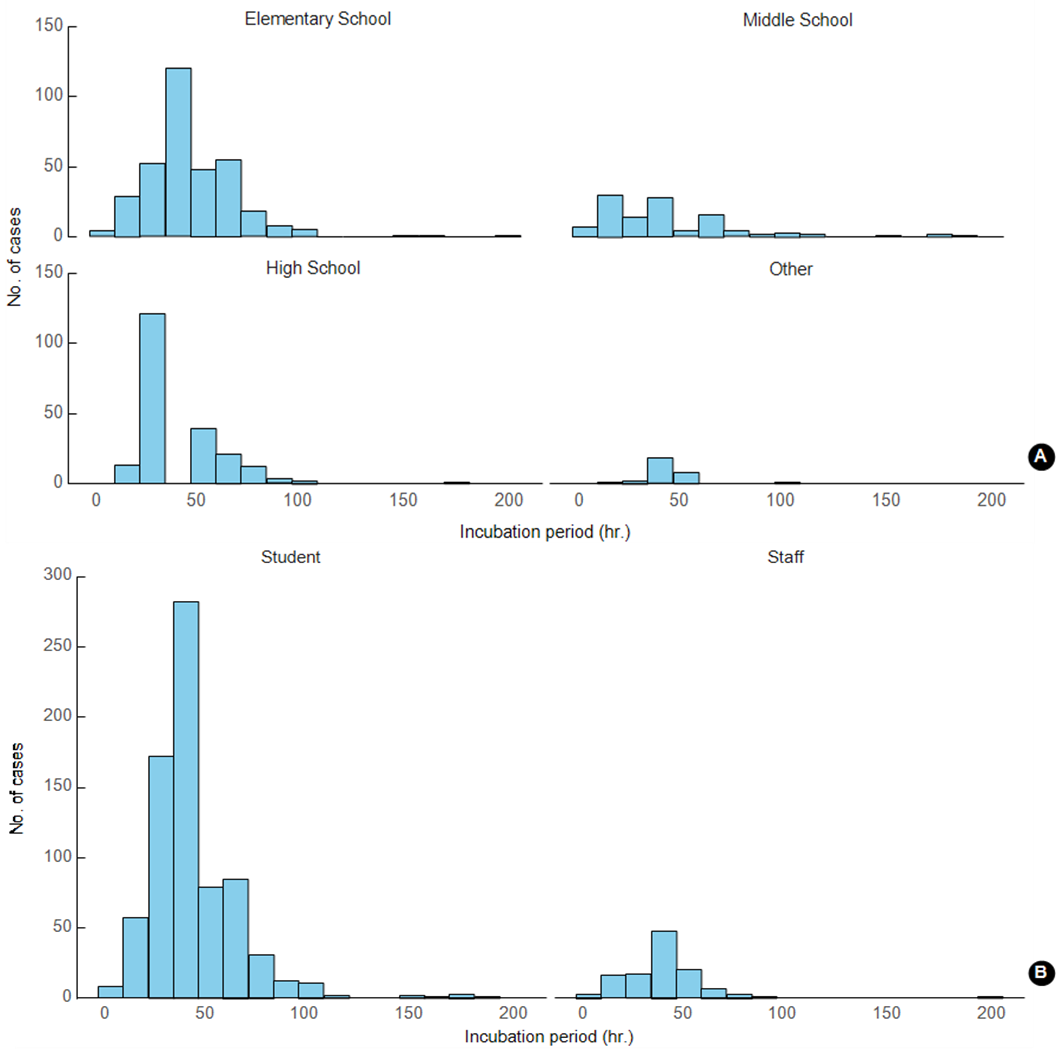

Supplement: Supplementary Material 1. — Distribution of incubation periods of cases of norovirus during the outbreak, stratified by (A) school type and (B) occupation [file epih-47-e2025057-Supplementary-1.docx]
